# Insanity and Crime

**Published:** 1924-01

**Authors:** 


### INSANITY AND CRIME.

THE recent Report of Lord Justice Atkin's Committee on the medico-legal aspects of insanity and crime has produced the inevitable fresh discussion of a subject which never slumbers for long. The outstanding recommendation of the Committee was that "uncontrollable impulse" should be accepted as one of the conditions rendering a criminal irresponsible for his crime. Upon this the lawyers and the doctors have once more joined issue, though there appear to be signs that, with the exception of the rigid, high and dry variety, the lawyers are somewhat more disposed than formerly to recognise that psychology may, after all, be a real aid to the administration of justice. The fact that the Medico-Legal Society is steadily growing in numbers is an indication that the importance of an understanding between law and medicine is becoming more clearly appreciated, although it is probable that the medical siege of the jury-box and the judgment-seat will never lead to an unconditional surrender. Nor is it desirable that it should—the psychological excuse for crime may very easily be carried too far.

The legal presumption, broadly stated, is that a person who commits a crime is responsible for his act unless he was clearly insane at the time of its commission. Since the crystallisation of that presumption we have discovered that the impulse to crime may, in fact, be uncontrollable even though, for the ordinary purposes of life, the criminal may be sane enough. The difficulty comes in when justice has to decide whether the degree of mental aberration, permanent or temporary, is sufficient to save the delinquent from the ordinary legal consequences of his offence. Whenever this question arises in an acute form, as in the case of Ronald True, and perhaps in that of the taxi-cab driver who has just been sentenced to death for an apparently purposeless murder, somebody has got to decide whether the legal or the medical view is to prevail. Mr. Shortt, the late Home Secretary, has just been stating his conviction that in such cases the last word ought to rest with the doctors. He instanced the condemnation of True because he did not come within the legal definition of insanity, whereas a Medical Commission decided that he was mad and therefore not in a condition to be hanged.

Legal history is full of the abandonment of presumptions of law upon all manner of subjects, and sooner or later we shall undoubtedly have to abandon the presumption that when a man murders, or commits some other serious crime, he is unquestionably sane. But we must needs move very cautiously. The choice between hanging an insane murderer and running the risk of seriously weakening the criminal law is a hard one, but it may sometimes have to be faced. After all, murder is murder, and society cannot afford to weaken its safeguards against it. Yet it is obvious that scientific justice requires that due weight shall be given to the plea of impaired control. That is already a familiar defence, and it is certain to become more and more common. It must necessarily make the work of judge and jury more difficult, since they will have to decide between the facts as proved in evidence, and the opinions, sometimes diverse, of medical men taken in conjunction with the demeanour of the prisoner. To lay down any fresh rule of law on the subject is obviously exceedingly difficult. Human aberrations cannot readily be made to fit into a formula, and it is the province of justice to discriminate, however difficult the task may be. To bring the rigour of the law upon the head of the fool or the mentally decayed would be a gross travesty of justice, yet we have always to be upon our guard against the ingenuity of those who simulate irresponsibility. As time passes and experience is gained the position will, no doubt, become clarified, and in the meantime we cannot do better than follow Mr. Shortt's suggestion that medical men should have every opportunity of instructing prison officials in the best way of dealing with prisoners whose minds are more or less clouded.

